# Higher baseline blood glucose is associated with reduced likelihood for successful recanalization in patients with basilar artery occlusion

**DOI:** 10.1007/s00415-021-10948-1

**Published:** 2022-01-04

**Authors:** Gabriel Broocks, Maximilian Groffmann, Lukas Meyer, Sarah Elsayed, Helge Kniep, Andre Kemmling, Noel van Horn, Rosalie McDonough, Tobias D. Faizy, Matthias Bechstein, Peter Sporns, Thilo Rusche, Gerhard Schön, Jawed Nawabi, Jens Fiehler, Uta Hanning

**Affiliations:** 1 Department of Diagnostic and Interventional Neuroradiology, University Medical Center Hamburg-Eppendorf, Martinistrasse 52, 20246 Hamburg, Germany; 2 Department of Neuroradiology, Westpfalz-Klinikum, Kaiserslautern, Germany; 3 Department of Neuroradiology, University Hospital Marburg, Marburg, Germany; 4 Department of Neuroradiology, Universitätsspital Basel, Basel, Switzerland; 5 Department of Radiology, University Hospital Münster, Münster, Germany; 6 Institute of Medical Biometry and Epidemiology, University Medical Center Hamburg-Eppendorf, Hamburg, Germany; 7 Department of Radiology, Charité University Medical Center, Berlin, Germany; 8 Berlin Institute of Health (BIH), BIH Biomedical Innovation Academy, Berlin, Germany

**Keywords:** Stroke, Ischemia, Infarction, Thrombolysis, Computed Tomography

## Abstract

**Purpose:**

Evidence regarding the effect of mechanical thrombectomy (MT) of basilar artery occlusion (BAO) stroke is yet sparse. As successful recanalization has been suggested as major determinant of outcome, the early identification of modifiable factors associated with successful recanalization could be of importance to improve functional outcome. Hyperglycemia has been associated with enhanced thrombin generation and unfavorably altered clot features.

**Objective:**

We hypothesized that serum baseline glucose is associated with likelihood of vessel recanalization mediated by collateral quality and clot burden in BAO stroke.

**Methods:**

BAO stroke patients who received multimodal CT on admission were analyzed. The association of vessel recanalization defined using modified Thrombolysis in cerebral infarction scale (mTICI) scores 2b-3, and baseline imaging and clinical parameters were tested in logistic regression analyses. Collateral quality and clot burden were evaluated using the Basilar Artery on CT-Angiography (BATMAN) score.

**Results:**

Out of 117 BAO patients, 91 patients (78%) underwent MT. In 70 patients (77%), successful recanalization could be achieved (mTICI 2b/3). In multivariable logistic regression analysis, only a higher BGL (aOR 0.97, 95% CI 0.96–0.99, *p *= 0.03) and higher BATMAN score (aOR 1.77, 95% CI 1.11–2.82, *p *= 0.02) were independently associated with vessel recanalization. Application of alteplase, or time from symptom onset-imaging revealed no independent association with recanalization status.

**Conclusion:**

Higher BGL was significantly associated with reduced likelihood for recanalization success besides BATMAN score as a measure of collateral quality and clot burden. BGL could be tested as a modifiable parameter to increase likelihood for recanalization in BAO stroke, aiming to improve functional outcome.

**Supplementary Information:**

The online version contains supplementary material available at 10.1007/s00415-021-10948-1.

## Introduction

Mechanical thrombectomy (MT) is associated with improved functional outcome when performed in patients with ischemic stroke and large vessel occlusion in the anterior circulation [11, 20, 27]. For patients presenting with an acute occlusion of the basilar artery (BAO), however, evidence is yet spare, although this patient population represents about 20% of all stroke patients [12]. Successful vessel recanalization has been described as the most important predictor of good functional outcome in BAO stroke [7]. Accordingly, failed endovascular recanalization was associated with a 13-fold increase of risk for poor outcome. Hence, the early identification of factors, especially modifiable factors, associated with successful recanalization could be of clinical importance to improve functional outcome in BAO patients.

In patients with acute coronary syndrome, prior studies observed that elevated glucose levels are associated with enhanced thrombin formation, platelet activation, and fibrin clot resistance [28]. For patients with ischemic stroke, it has been described that hyperglycemia may increase infarct volume, risk for secondary hemorrhage, and finally lead to a worse prognosis [17, 18]. Furthermore, observations in alteplase-treated patients confirmed that acute hyperglycemia may impede the fibrinolytic process potentially affecting reperfusion. Hence, it has been concluded that early measures to reduce elevated blood glucose levels could improve early recanalization [19]. Yet, the impact of baseline serum glucose levels (BGL) on recanalization success, especially in BAO stroke patients, remains uncertain. While acknowledging a history of diabetes mellitus as potential factor, most previous studies investigating reasons for unsuccessful recanalization did not specifically analyze BGL [10, 13]. A recent study observed that diabetes mellitus was associated with an increased likelihood of a first pass effect [14]. That raises the question, whether patients with known diabetes treated adequately could exhibit lower glucose levels on admission, compared to (often elderly) patients with pre-diabetes or undiagnosed diabetes and subsequent higher baseline BGL. Anti-diabetic treatment might also have an impact on the likelihood of reperfusion, and hence better functional outcome. In line with this, a previous study observed that stroke patients medicated with sulfonylureas had better outcomes [16]. Yet, it has not been investigated whether BGL is associated with likelihood of recanalization. Better knowledge of the role of BGL in BAO stroke may be of high clinical relevance, considering that in contrast to anterior circulation stroke, diagnosis is often delayed and reperfusion rates are very low in the absence of MT (i.e. 4% [2]) [7, 8].

The purpose of this study was to investigate factors associated with successful recanalization in BAO stroke, in particular BGL. As the status of intracranial collaterals might mediate the effect of BGL, we also sought to investigate the relationship of BGL and collateral quality and its combined diagnostic accuracy to predict vessel recanalization. We hypothesized that BGL are associated with likelihood of recanalization.

## Methods

### Patient cohort

All patients admitted with acute ischemic posterior circulation stroke between June 2015 and February 2019 at two high-volume tertiary stroke centers were retrospectively analyzed. Anonymized data were recorded and analyzed in accordance with ethical guidelines and after approval of the local ethics committee (IRB approval number WF04/13). Informed consent was waived.

The inclusion criteria for this study were: (1) acute ischemic posterior circulation stroke with multimodal CT imaging on admission [non-enhanced CT (NECT), CT-angiography (CTA) and CT perfusion (CTP)]; (2) occlusion of the basilar artery apparent on CTA; (3) last known well < 24 h; (4) absence of intracranial hemorrhage; (5) absence of significant imaging artifacts.

Successful endovascular recanalization was defined by modified Thrombolysis in Cerebral Infarction (mTICI)-classification as mTICI score ≥ 2b at the conclusion of the procedure by the performing neurointerventionalist, validated by a further attending neuroradiologist.

### Image analysis

To assess the degree of early ischemic changes, Posterior circulation Acute Stroke Prognosis Early CT score (pcASPECTS) was rated by two experienced board-certified neuroradiologists, and discrepancies were settled by joint discussion of both readers. The collateral quality and clot burden was evaluated using the Basilar Artery on CT-Angiography (BATMAN) score, a semiquantitative CTA-based grading system [1]. Consequently, the vertebrobasilar system was divided into six segments [vertebral arteries = 1, basilar artery = 2–4, posterior cerebral artery, PCA = 5, posterior communicating artery (Pcom) = 6]. Points were allocated, as described: 2 points for each Pcom, 1 point for a hypoplastic Pcom (< 1 mm) if in continuity with the basilar artery top via P1-posterior cerebral artery (PCA), or 3 points for each fetal Pcom, 1 point for each of the other segments (= maximum score of 10). Rating of BATMAN was performed by two experienced board-certified neuroradiologists who were blinded to the scores of the other reader. Discrepancies about the BATMAN score were settled by joint discussion of the two readers.

### Image acquisitions

All included patients received a comprehensive stroke imaging protocol at admission with NECT and CTA and additional CTP consecutively in equal order on an iCT 256^™^ scanner (Philips Healthcare, Best, The Netherlands). NECT: collimation 64 × 0.625, pitch 0.297, rotation time 0.4 s, FOV 270 mm, tube voltage 120 kV, tube current 300 mA, 4.0 mm slice reconstruction. CTA: collimation 64 × 0.625, pitch 0.985, rotation time 0.4 s, FOV 220 mm, tube voltage 120 kV, 300 mAs, 2.0 mm slice reconstruction, 5 mm MIP reconstruction with 1 mm increment. CTP: collimation 64 × 1.25, rotation time 0.5 s, FOV 220 mm, tube current 80 kV, tube current 140 mAs, 5 mm slice reconstruction, slice sampling rate 1.8 s, scan time 72 s, biphasic injection with 40 ml of highly iodinated contrast medium with 400 mM/ml injected with 6 ml/s followed by 40 ml NaCl chaser bolus.

### Statistical analysis

Categorial variables were compared using *χ*^2^ tests. Distribution of metric variables was described by means and standard deviation (SD) or medians and interquartile ranges (IQR). Kolmogorov–smirnov tests were used to test for normal distribution. Student *t* tests (normal distribution) with SD or Mann–Whitney-*U* tests (non-normal distribution) with IQR were used to determine differences of the acquired parameters for patients with versus without recanalization.

We defined a binary outcome (successful recanalization) as mTICI score ≥ 2b. Logistic regression analysis was performed to assess the association between the clinical and radiological parameters, and outcome (successful recanalization versus no successful recanalization). For univariable logistic regression analysis, all available independent variables (BGL, BATMAN score, time from onset to imaging, age, sex, application of alteplase, NIHSS on admission, and pcASPECTS) were analyzed with successful recanalization as dependent variable (Table 2). Second, multivariable logistic regression models were performed implementing the independent variables that showed a significant association in univariable analysis. Correlation between all independent variables was tested to exclude multicollinearity. The area under the curve for the resulting logistic model was calculated to show its diagnostic ability. The probability for vessel recanalization according to the BATMAN score was plotted separately for patients with different levels of BGL (trichotomized based on 25% and 75% percentile). Finally, it was analyzed whether the aforementioned variables and recanalization status are associated with functional outcome assessed using uni- and multivariable ordinal regression analysis with modified Rankin Scale (mRS) scores at day 90 as dependent variable.

A statistically significant difference was accepted at a *p* value of less than 0.05. Analyses were performed using MedCalc (version 11.5.1.0; Mariakerke, Belgium) and Stata 13.0 (StataSE, StataCorp, TX, USA).

## Results

### Patients

**Fig. 1:**
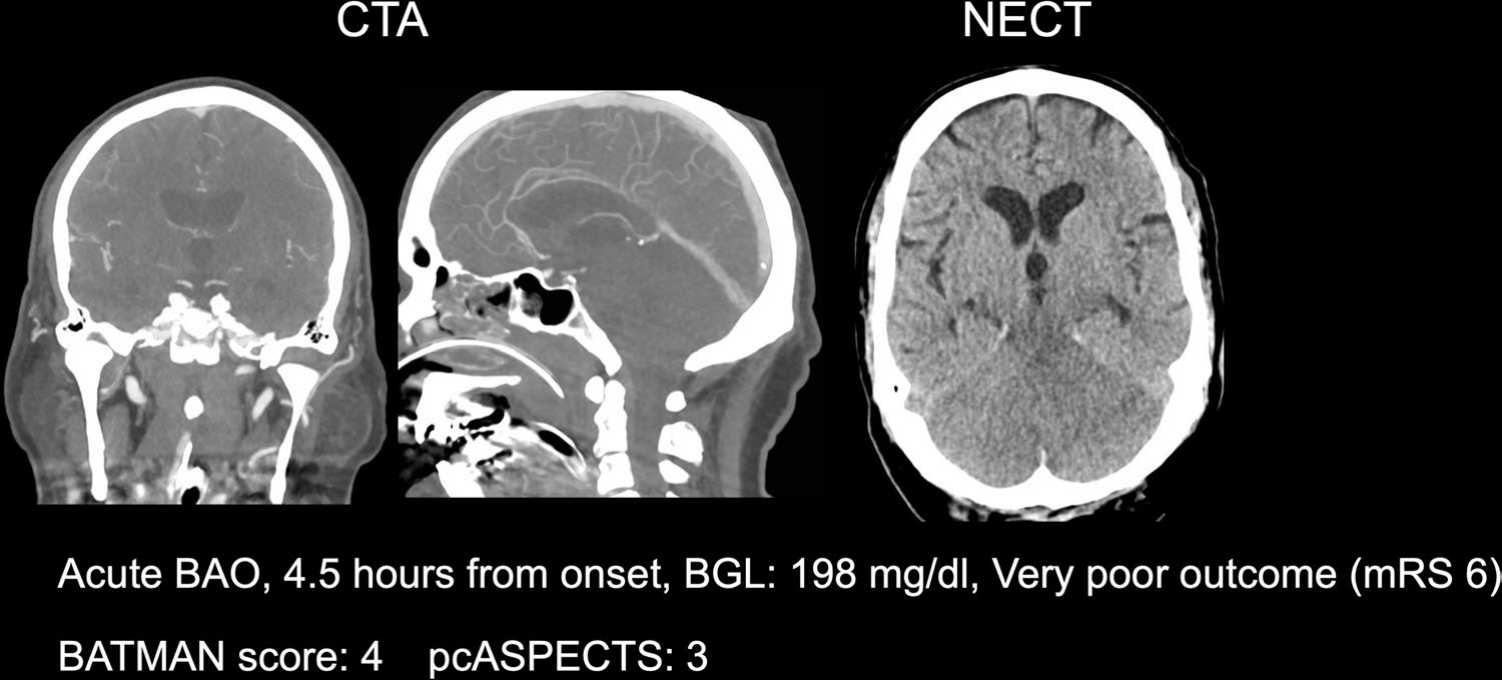
Example of a patient with low BATMAN score and high BGL. Illustration of a patient with acute basilar artery occlusion, poor BATMAN score, and high BGL on admission

The patient characteristics including baseline, treatment, and outcome variables are displayed in Table 1, separately for patients with successful recanalization versus patients without successful recanalization. Figure 1 shows an example of a patient with high BGL and low BATMAN score. A patient inclusion flow chart is included in the supplemental material. A total of 117 patients were included. The mean age was 69 years (SD 13.6) and 46 patients were female (39%). In 82 patients (70%), time from symptom onset to imaging was known. In these patients, the median time from symptom onset to admission imaging was 3.3 h (IQR 1.3–7.2 h). 35 patients (29%) had an unknown onset (within 24 h from last known well). In total, 91 patients underwent MT (77%), of which 70 (77%) resulted in successful recanalization (mTICI 2b/3). Among 35 patients with unknown onset, 30 patients underwent MT (86%), and 60 patients (73%) from patients with known time window underwent MT (*p *= 0.14). The median NIHSS on admission was 20 (IQR 8–42). The median pcASPECTS was 9 (IQR 8–9) and the median BATMAN score was 7 (IQR 5–9). The median BGL was 134 mg/dl (IQR 115–160). In 23 patients (20%) a known history of diabetes mellitus type 2 was deducted from medical documentation. The median mRS score at day 90 was 4 (IQR 1–5). Table 1 displays the patient characteristics.

**Table 1:** Patient characteristics

	MT with mTICI 2b-3 *n* = 70	No MT/MT with mTICI 0-2a *n* = 47	*p* value
Baseline characteristics			
Age in years, mean (SD)	68 (1.8)	71 (1.5)	0.21
Sex female, *n* (%)	25 (36)	21 (44)	0.41
Known time from onset to imaging in hours, median (IQR)	2.5 (1.2–6.0)	4.0 (2.0–12.0)	0.04
Unknown onset within 24 h, *n* (%)	24 (35)	11 (23)	0.17
NIHSS on admission, median (IQR)	22 (12–42)	18 (4–36)	0.09
Glucose, median mg/dl (IQR)	126 (111–149)	144 (120–177)	0.01
History of Diabetes, *n* (%)	14 (20)	9 (18)	0.21
BATMAN score, median (IQR)	7 (6–9)	5 (5–7)	0.02
pcASPECTS, median (IQR)	9 (8–10)	9 (8–9)	0.07
Treatment and follow-up			
IV alteplase, *n* (%)	38 (55)	17 (29)	0.04
NIHSS at discharge, median (IQR)	3 (1–15)	10 (3–23)	0.09
mRS after 90 days, median (IQR)	4 (1–5)	5 (1–6)	0.19

*IQR* indicates interquartile range, *NIHSS* National Institute of Health Stroke Scale, *pcASPECTS* posterior circulation Alberta Stroke Program Early CT Score, *IV* intravenous, *TICI* thrombolysis in cerebral infarctions, *mRS* modified Ranking Scale

Comparing patients with successful recanalization to patients without, there was no significant difference in age, sex, or NIHSS on admission (*p *= 0.21/0.41/0.09). Likewise, the rate of patients with unknown onset within 24 h from last known well was not different, although patients with successful recanalization offered a shorter time from known onset of stroke symptoms (2.5 h versus 4.0 h, *p *= 0.04). Moreover, patients with successful recanalization had a significantly higher BATMAN score (7 versus 5, *p *= 0.02), lower BGL (126 mg/dl versus 144 mg/dl, *p *= 0.01), and received intravenous alteplase more frequently (55% versus 29%, *p *= 0.04) (Table 1).

### Prediction of successful recanalization

For this analysis, only patients undergoing MT (*n* = 91) were included. There was no significant correlation between the independent variables (*r* <  ± 0.2). In univariable logistic regression analysis, BGL, BATMAN score, intravenous alteplase, and time from onset to imaging were significantly associated with successful recanalization (Table 2). No association between age, sex, pcASPECTS, or NIHSS and recanalization was observed.

**Table 2:** Binary logistic regression analysis for successful recanalization (TICI 2b/3)

	Univariable analysis^a^			Multivariable analysis		
	OR	95% CI	*p* value	OR	95% CI	*p* value
Time onset-imaging	0.92	0.86–0.99	0.03	0.89	0.75–1.06	> 0.4
BGL	0.98	0.98–0.99	0.01	0.98	0.95–0.99	0.03
BATMAN	1.51	1.07– 2.13	0.02	1.87	1.07–3.29	0.03
IV alteplase	2.24	1.05–4.78	0.04	1.12	0.13–4.79	> 0.3
Age	0.98	0.95–1.01	0.21	–	–	–
Sex	0.73	0.34–1.55	0.41	–	–	–
NIHSS	1.02	0.99–1.04	0.18	–	–	–
pcASPECTS	1.19	0.94–1.50	0.14	–	–	–

^a^Age, Sex, NIHSS on admission, and pcASPECTS were not significantly associated with recanalization in univariable analysis

**Fig. 2:**
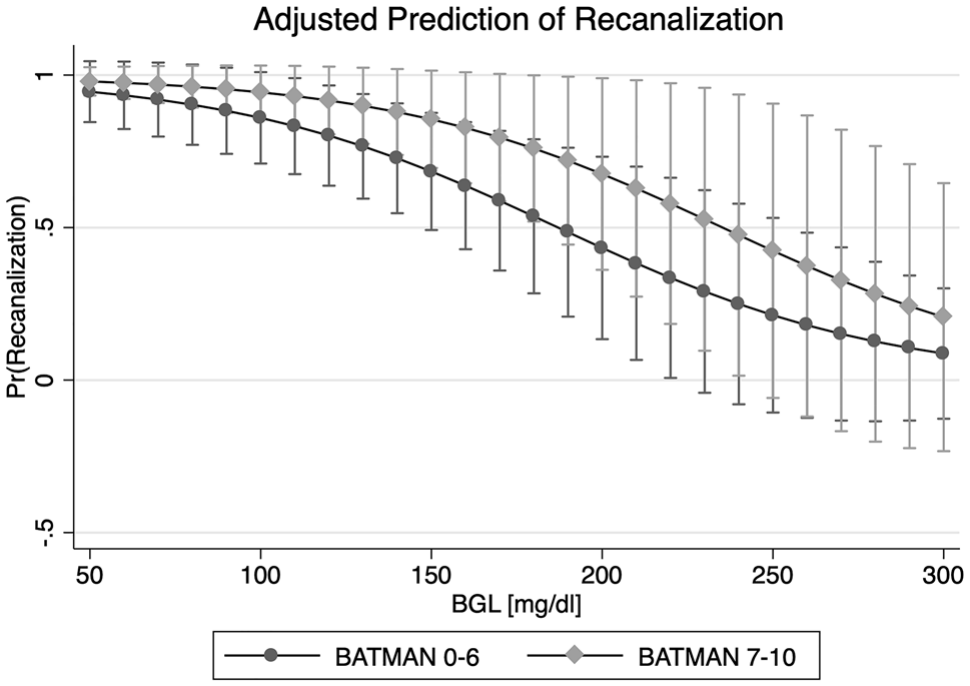
Association of baseline glucose level and dichotomized BATMAN score on the likelihood for recanalization. Multivariable logistic regression plot to illustrate the impact of glucose (*x* axis, in mg/dl) and BATMAN score (dichotomized) on the likelihood of successful recanalization

**Fig. 3:**
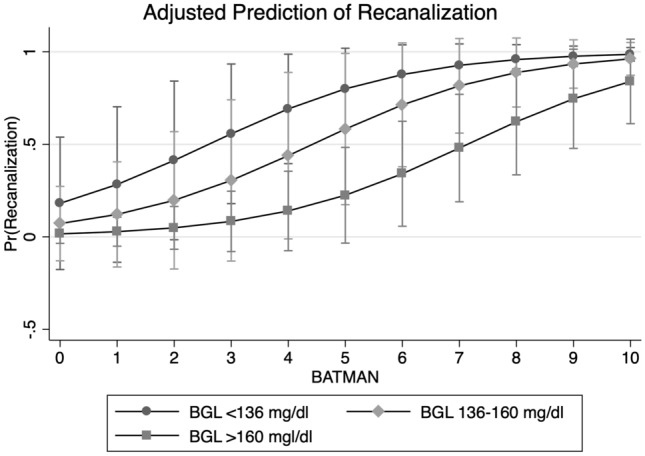
Association of BATMAN score and trichotomized baseline glucose level on likelihood for recanalization. Multivariable logistic regression plot to illustrate the impact of the BATMAN score (*x* axis) and trichotomized blood glucose level (BGL) on the likelihood of successful recanalization

In multivariable logistic regression analysis, BGL (OR 0.98, 95% CI 0.95–0.99, *p *= 0.03) and BATMAN (OR 1.87, 95% CI 1.07–3.29, *p *= 0.03) remained the only variables independently associated with successful recanalization (Fig. 2, Table 2). The diagnostic ability to classify recanalization status of this predictive model based on the area under the ROC curve was excellent (AUC 0.91). Figure 3 shows the incremental change of likelihood for recanalization based on the BATMAN score separately for patients with low (< 25% percentile, < 136 mg/dl) intermediate (25–75% percentile, 136–160 mg/dl, and high BGL (> 75% percentile, > 160 mg/dl).

### Prediction of functional outcome

**Fig. 4:**
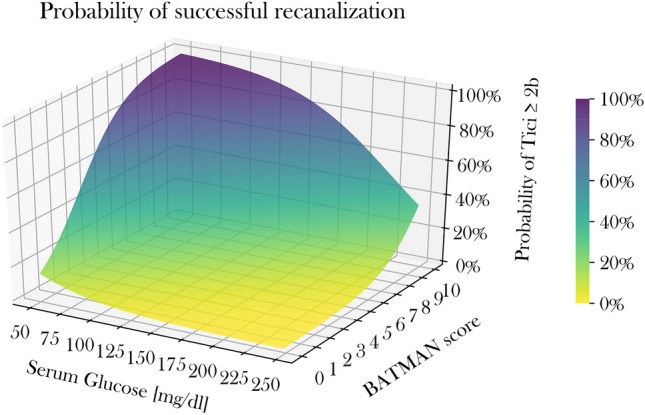
3D Illustration of the relationship of glucose levels and BATMAN score on the likelihood for recanalization. 3D Surface plot to illustrate the impact of BATMAN scores and BGL on the likelihood for successful recanalization

Ordinal regression analysis was performed to identify variables associated with functional outcome at day 90. In univariable ordinal regression analysis, higher BGL was by trend associated with a higher mRS at day 90 (OR 1.007, 95% CI 0.999–1.013, *p *= 0.06). Successful recanalization was not associated with functional outcome (*p *> 0.1). After adding BATMAN, and BGL to the model, however, successful recanalization was significantly associated with functional outcome (OR for mRS shift: 0.18, 95% CI 0.04–0.78, *p *= 0.02). In multivariable ordinal regression analysis, vessel recanalization, pcASPECTS, and NIHSS were independently associated with clinical outcome, adjusted for BATMAN, BGL, intravenous alteplase, and age (Table 3) (Fig. 4).

**Table 3:** Uni- and multivariable ordinal regression analysis for clinical outcome (mRS shift)

	Univariable analysis			Multivariable analysis		
	OR	95% CI	*p* value	OR	95% CI	*p* value
BGL	1.00	0.99–1.01	0.06	1.01	0.99–1.02	> 0.3
BATMAN	0.84	0.66–1.08	0.17	1.14	0.86–1.52	> 0.3
Recanalization	0.65	0.34–1.25	0.20	0.18	0.04–0.84	0.03
NIHSS	1.08	1.06–1.11	< 0.001	1.05	1.00–1.10	0.04
IV alteplase	1.48	0.78–2.82	0.23	1.10	0.30–4.00	> 0.8
pcASPECTS	0.72	0.58–0.89	0.002	0.58	0.39–0.86	< 0.01
Age	1.03	1.00–1.06	0.01	1.04	0.38–0.86	0.08
Time	0.98	0.96–1.00	0.14	1.07	0.92–1.24	> 0.5

*BGL* baseline glucose level, *Time* Time from symptom onset to admission

## Discussion

The aim of this study was to investigate factors associated with successful recanalization in acute BAO patients, and in particular, analyze how baseline BGL and the BATMAN score, as a measure of collateral quality and clot burden, are associated with reperfusion. We hypothesized that BGL is directly related to the likelihood of successful recanalization, mediated by the BATMAN score. The study has several findings: (1) in multivariable logistic regression analysis, only BGL and BATMAN were independently associated with recanalization; (2) successful vessel recanalization was significantly associated with better functional outcome when adjusted for BGL and BATMAN score; (3) pcASPECTS and recanalization status were important predictors of functional outcome, while other variables such as application of intravenous alteplase, or time from onset to imaging, only had a minor impact on recanalization, and functional outcome.

In ischemic posterior circulation stroke, BAO is still associated with very poor functional outcome, despite recent advances in endovascular treatment. Lately, the results of the Basilar Artery International Cooperation Study (BASICS), analyzing the safety and efficacy of MT in BAO patients, have been presented (clinical trial ID: NCT01717755 [23]). This study observed no significant benefit of MT compared to best medical management alone, notwithstanding a slightly higher proportion of patients with good functional outcome. Despite controversies about the study design and particularly its long recruitment period, the study emphasizes a need to further improve treatment selection for MT and outcome prediction in BAO stroke.

Successful endovascular recanalization has been described as the most important predictor of good clinical outcome following BAO stroke, while failed thrombectomy was associated with a significant increase of risk for poor outcome [7, 9]. Therefore, the early identification of factors, in particular factors that can be adjusted in the acute situation, associated with successful recanalization might be of significant importance to improve functional outcome. In this study, only the BATMAN score, and BGL were independently associated with successful vessel recanalization. While it has been observed that elevated BGL are associated with worse clinical outcome, it has not yet been described whether BGL may influence the likelihood of endovascular recanalization. A recently published study showed that higher glucose levels on admission are independent predictors of failure of early neurological improvement (“fENI”) after successful thrombectomy [29]. Moreover, patients with anterior circulation stroke and higher glucose levels showed worse outcome, but the effect of hyperglycemia was exacerbated in patients with incomplete MT. That leads to the question, whether hyperglycemia could reduce likelihood for successful MT, and hence be associated with worse outcomes [15]. Previous studies investigating the impact of BGL on outcome in patients with acute coronary syndrome observed that elevated glucose levels are associated with enhanced thrombin formation, platelet activation, and fibrin clot [28]. In acute ischemic stroke, previous studies described that hyperglycemia may increase infarct volume, risk for secondary hemorrhage, and finally lead to a poor prognosis and that hyperglycemia could impede the fibrinolytic process potentially affecting reperfusion [17, 19]. A further clinical trial evaluated the effects of hyperglycemia on recanalization rates and outcome in patients receiving intravenous alteplase, and the authors concluded that higher serum glucose was associated with lower rates of recanalization and worse outcome [22]. The question remained whether strict control of BGL might raise the likelihood for reperfusion. It is, however, important to realize that recanalization of BAO after intravenous alteplase alone occurs rarely. A prior study observed that only 4% of BAO patients may show recanalization after alteplase alone [2]. Therefore, the aforementioned observations on patients receiving alteplase-only cannot be directly related to patient cohorts undergoing thrombectomy. In contrast to BGL and BATMAN score, there was no independent association between the application of intravenous alteplase, or time from symptom onset to imaging and recanalization. Furthermore, it remains uncertain whether persistent higher BGL or acute BGL increases are causative and to what extent. Future research should include HbA1c and serial BGL measurements to elucidate this coherence.

The second prognostic factor that was associated with likelihood of recanalization was the BATMAN score, determined in baseline CTA. CT is the most widely performed imaging modality in acute stroke, and CTA the standard approach to diagnose BAO, allowing the noninvasive assessment of the collateral status, which might have an important impact on the outcome of BAO patients. The BATMAN score takes into account both the extent of the occlusion (i.e. number of obstructed perforating arteries and therefore extent of ischemia), and the presence of Pcom arteries that are known to be associated with a better prognosis in BAO [1]. It has been described that the additional assessment of collateral quality to clot burden improves the diagnostic accuracy in BAO stroke [1].

To our knowledge, this is the first study that investigated baseline variables associated with successful recanalization in BAO, and the first study to analyze how BGL directly affects the likelihood for vessel recanalization, depending on the collateral status, and clot burden. Analyzing factors associated with successful recanalization in BAO has particular clinical relevance considering the very poor outcomes associated with incomplete or failed MT and the comparably lower effect of intravenous alteplase (i.e. recanalization rate approximately 4% in BAO compared to approximately 30% in middle cerebral artery occlusion) [2, 7, 8].

In accordance with a previous study, we observed in multivariable analysis that successful vessel recanalization is independently associated with better functional outcome (see Table 3) [7]. Besides recanalization status, pcASPECTS was an important predictor of outcome, although not associated with recanalization status. Nevertheless, pcASPECTS should be taken into account in the assessment of posterior circulation stroke patients. Patients with a low pcASPECTS are at particular risk of developing malignant cerebellar edema and should be monitored closely [3, 21].

In the clinical context, the assessment of the BATMAN score and acknowledgement of BGL might help to estimate the likelihood of recanalization, or even consider insistent normalization of BGL to improve chances for successful thrombectomy. A desirable side effect of BGL adjustment might be the reduction of ischemic edema, as recently described [5], which might have a further positive effect on functional outcome. Currently, anti-diabetic drugs are tested as adjuvant treatment option in ischemic stroke, such as Glyburide, an inhibitor of the sulfonylurea receptor-1, and transient receptor potential melastatin 4 (SUR1-TRPM4). It has been reported that glyburide might lower formation of ischemic brain edema, subsequently decreasing infarct volume, reducing mortality, and improving outcomes [16, 24, 26]. Edema reduction could constitute an interesting therapeutic target, as space-occupying edema is regularly associated with devastating outcomes [4]. Despite several previous studies testing neuroprotectants and therapeutic BGL adjustment to improve outcome in stroke, the subjects included in these studies did mostly not achieve reperfusion, which could significantly limit the effects of adjuvant treatment options [6, 25].

The results of the present study advocate the further implementation of imaging tools in the assessment of posterior circulation stroke patients in daily clinical practice, such as pcASPECTS, and the BATMAN score, as easy and fast methods to gain more insights into pathophysiological processes, which could be helpful particularly in cases with uncertain time window and/or limited clinical assessability.

There are several limitations of this study. First, this was a retrospective study with a limited number of patients and risk of selection bias. Furthermore, hyperglycemia during ischemic stroke is a dynamic process, and single assessments of BGL might not be sufficient to capture the complexity. Further studies could assess multiple measurements of BGL and analyze the impact of HbA1c values that were not available in the present study, because they are not part of the routine laboratory for acute ischemic stroke patients. Higher BGL on admission might be accompanied by further unfavorable metabolic epiphenomena, and cardiovascular risk factors, leading to a potential bias. Finally, information on previous medication was not available. Future research is needed to investigate the potential impact of antidiabetic medication.

## Conclusion

In this study, lower BGL and the higher BATMAN scores were the only independent predictors of successful vessel recanalization in acute BAO, which was associated with better functional outcome at day 90. BGL might constitute a modifiable variable to increase likelihood for successful treatment, and the impact of BGL adjustments might be tested in further prospective studies.

## Supplementary Information

Below is the link to the electronic supplementary material.

Supplementary file1 (DOCX 170 KB)

## Data Availability

The data that support the findings of this study are available from the corresponding author upon reasonable request.

## Abbreviations

CT: Computed tomography
MT: Mechanical thrombectomy
BATMAN: Basilar artery on CT-angiography
NIHSS: National Institute of Health Stroke Scale
BAO: Basilar artery occlusion
NECT: Non-enhanced computed tomography
mTICI: Modified Thrombolysis in cerebral infarction scale
BGL: Baseline serum glucose levels
pcASPECTS: Posterior circulation Acute Stroke Prognosis Early CT score
