# Supplementary figures and images for: Higher baseline blood glucose is associated with reduced likelihood for successful recanalization in patients with basilar artery occlusion
Source: J Neurol. 2022 Jan 4;269(6):3286–94. doi: 10.1007/s00415-021-10948-1 (PMC9120087; doi:10.1007/s00415-021-10948-1)

***ONLINE SUPPLEMENT***

***Flow Chart of Patient Inclusion:***


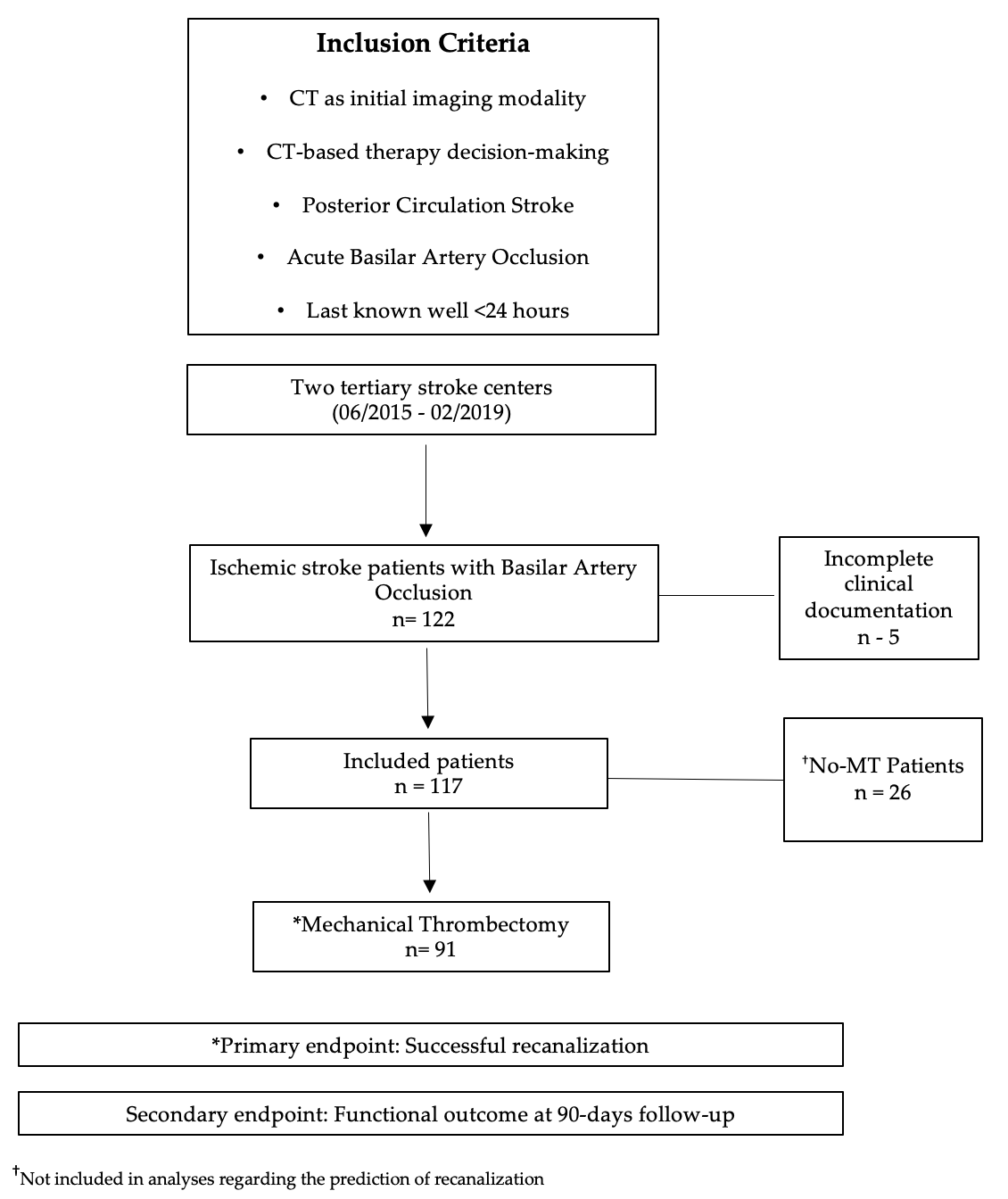

Supplement: Supplementary file 1 — Supplementary file1 (DOCX 170 KB) [file 415_2021_10948_MOESM1_ESM.docx]
